# The complete mitochondrial genome of the deep-sea amphipod *Eurythenes magellanicus* (Crustacea: Amphipoda: Lysianassidae)

**DOI:** 10.1080/23802359.2019.1703573

**Published:** 2019-12-17

**Authors:** Jun-yuan Li, Yan-wen Liao, Jun Li, Li-sheng He

**Affiliations:** a Institute of Deep-sea Science and Engineering, Chinese Academy of Sciences, Sanya, China;; b College of Earth and Planetary Sciences, University of Chinese Academy of Sciences, Beijing, China

**Keywords:** Amphipod, deep sea, mitochondrial genome

## Abstract

The complete mitochondrial genome of the deep sea amphipod *Eurythenes magellanicus* was determined in this paper. This molecular was 14,988 bp in length, and contained the typical 13 protein coding genes (PCGs), 22 transfer RNAs (tRNAs), two ribosomal RNAs (rRNAs) and one control region (CR). The gene order of *E. magellanicus* was identical to that from *E. maldoror*, a deep sea amphipod inhabiting in a deeper habitat than *E. magellanicus.* A maximum-likelihood tree based on the 13 PCGs from 25 amphipods indicated that *E. magellanicus* and *E. maldoror* were closely related and the origin of deep sea amphipods was not monophyletic.

The lysianassoid amphipods in genus *Eurythenes* are bathymetrically and geographically widespread in the ocean (Thurston 1990). They are one of the most abundant samples handily obtained by the bait trapper from the deep sea and have been used for many aspects of scientific researches including species diversity (Havermans et al. 2013), feed habits (Hargrave et al. 1995; Janßen et al. 2000), and life history characterization (Thurston and Bett 1995). However, several species in *Eurythenes* were overlooked so that they had been regarded as one taxa named *Eurythenes gryllus* (Bowman and Manning 1972; Ingram and Hessler 1987; Ichiro and Kentaro 1998; Janßen et al. 2000). In 2013, the differentiation of these cryptic species in the “*Eurythenes gryllus* complex” was confirmed with both morphological and molecular evidences (Havermans et al. 2013; Eustace et al. 2016) and the genus *Eurythenes* was classified into at least 15 species-level lineages (Havermans et al. 2013; Eustace et al. 2016; Havermans 2016). The depth could be the major factor resulting in their speciation (Eustace et al. 2016).

*Eurythenes magellanicus* represents one of the abyssal-major clades in *Eurythenes*. Most of *E. magellanicus* inhabits around 4400 m (Eustace et al. 2016), whereas two individuals have been collected at the bathyal depth of 1300 –1400 m (Havermans 2016; Narahara-Nakano et al. 2018). A total of four *E. magellanicus* specimens were collected at the depth of 1048 m (110°27.522'E 17°31.337'N) in the South China Sea in May 2019 by the baited trapper installed on our “Feng-huang” Lander, which consolidated the distribution of *E. magellanicus* in the bathyal ranges was not occasional. The samples were deposited in the specimen room of the protein research lab of the Institute of Deep-sea Science and Engineering, Chinese Academy of Sciences (accession no. Nanhai-20190523FH22-AMP1 to Nanhai-20190523FH22-AMP4). Here, the complete mitochondrial genome of *E. magellanicus* was determined. It would be helpful for the future phylogenetic study, especially for the *Eurythenes* group, within which there could still be a high diversity of cryptic species (Havermans 2016).

DNA extraction, high-throughput sequencing, sequence assembly, and gene annotation followed our previous procedures (Li, Zeng, et al. 2019; Li, Song, et al. 2019). The phylogenetic tree was constructed using the PhyloSuite1.1.15 pipeline (Zhang et al. 2019). The maximum-likelihood phylogeny was inferred using IQ-TREE 1.6.8. (Nguyen et al. 2017). mtZOA + F + I + G4 model was selected as recommended by the built-in ModelFinder module in IQ-tree (Chernomor et al. 2016).

The complete mitochondrial genome of *E. magellanicus* was 14,988 bp in length (NCBI accession no. MN688221) and had a typical component of 13 PCGs, 22 tRNAs, 2 rRNAs and a control region. The gene arrangement of *E. magellanicus* was identical to that of *Eurythenes maldoror* (accession no. NC036429), which was the only available mitochondrial genome from *Eurythenes* before the present study. Considering *E. maldoror* was a deep sea amphipod mainly distributed at the abyssal depth from 4000 m to 6000 m [11], the differentiation in *Eurythenes* caused by the depth stratification seemed to have no influence on the mitochondrial gene order.

Our constructed phylogeny indicated that *E. magellanicus* clustered with *E. maldoror*. These two *Eurythenes* species were grouped with the hadal amphipod *Hirondellea gigas* and the shallow water *Onisimus nanseni* in the Arctic. They belonged to the superfamily Lysianassoidea (Figure 1). Other superfamilies (Alicellidea and Dexaminoidea) also included taxa from the deep sea environment (Figure 1), therefore, the divergency for the deep sea adaptation happened multiple times during the evolution of Amphipoda.

**Figure 1.**
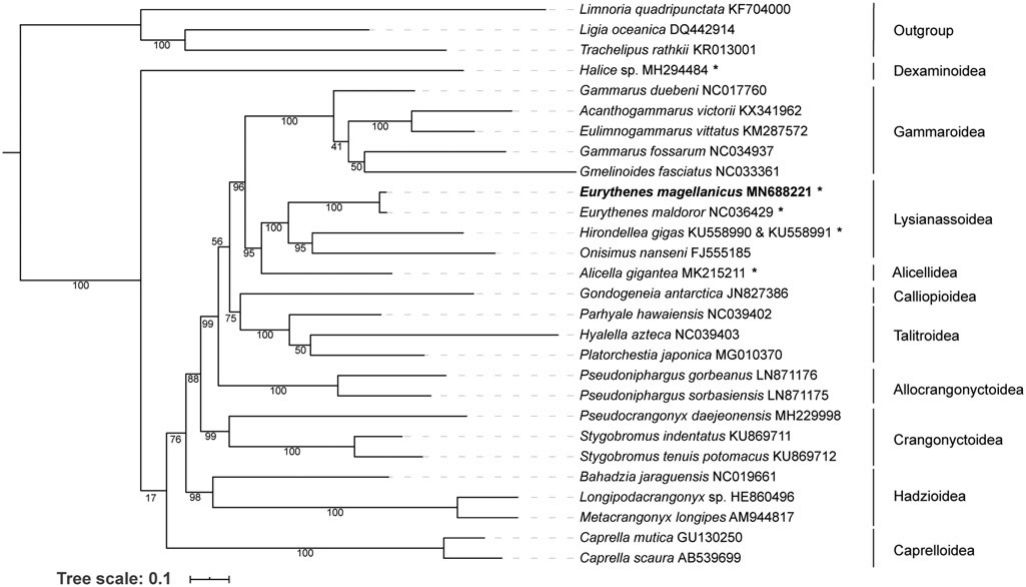
Phylogenetic tree based on the 13 protein coding genes of the mitochondrial genomes from *Eurythenes magellanicus* and other 24 amphipods in Amphipoda. Bootstraps values were shown at each node, Genbank numbers were after the species names. * indicated the samples were from the deep sea environment.
